# Recent Progress of RGD Modified Liposomes as Multistage Rocket Against Cancer

**DOI:** 10.3389/fphar.2021.803304

**Published:** 2022-01-25

**Authors:** Afsana Sheikh, Nabil A. Alhakamy, Shadab Md, Prashant Kesharwani

**Affiliations:** ^1^ Department of Pharmaceutics, School of Pharmaceutical Education and Research, Jamia Hamdard, New Delhi, India; ^2^ Department of Pharmaceutics, Faculty of Pharmacy, King Abdulaziz University, Jeddah, Saudi Arabia; ^3^ Center of Excellence for Drug Research and Pharmaceutical Industries, King Abdulaziz University, Jeddah, Saudi Arabia

**Keywords:** liposome, cancer, targeted therapy, integrin, RGD peptide, toxicity, nanomedicine

## Abstract

Cancer is a life-threatening disease, contributing approximately 9.4 million deaths worldwide. To address this challenge, scientific researchers have investigated molecules that could act as speed-breakers for cancer. As an abiotic drug delivery system, liposomes can hold both hydrophilic and lipophilic drugs, which promote a controlled release, accumulate in the tumor microenvironment, and achieve elongated half-life with an enhanced safety profile. To further improve the safety and impair the off-target effect, the surface of liposomes could be modified in a way that is easily identified by cancer cells, promotes uptake, and facilitates angiogenesis. Integrins are overexpressed on cancer cells, which upon activation promote downstream cell signaling and eventually activate specific pathways, promoting cell growth, proliferation, and migration. RGD peptides are easily recognized by integrin over expressed cells. Just like a multistage rocket, ligand anchored liposomes can be selectively recognized by target cells, accumulate at the specific site, and finally, release the drug in a specific and desired way. This review highlights the role of integrin in cancer development, so gain more insights into the phenomenon of tumor initiation and survival. Since RGD is recognized by the integrin family, the fate of RGD has been demonstrated after its binding with the acceptor’s family. The role of RGD based liposomes in targeting various cancer cells is also highlighted in the paper.

## Introduction

Since the discovery of liposomes in the 1960s they have contributed to a growth in the drug development industry owing to their unique characteristics such as bio-degradability, biocompatibility, lack of immune system activation, low toxicity, and capability to incorporate both hydrophobic and hydrophilic drugs. Liposomes are nanometric phospholipid bubbles with a lipid bilayer which, after incorporation of lipophilic/hydrophilic drug molecules, prevents the rapid degradation of the drug and also reduces toxicity (Torchilin, 2005; Bulbake et al., 2017). Using the specialized advantages of liposomes, the therapeutic window of potent drug molecules can be expanded by increasing metabolism and absorption, reducing the elimination rate, and promoting the biological half-life (Estanqueiro et al., 2015; Kesharwani et al., 2021).

**FIGURE 1:**
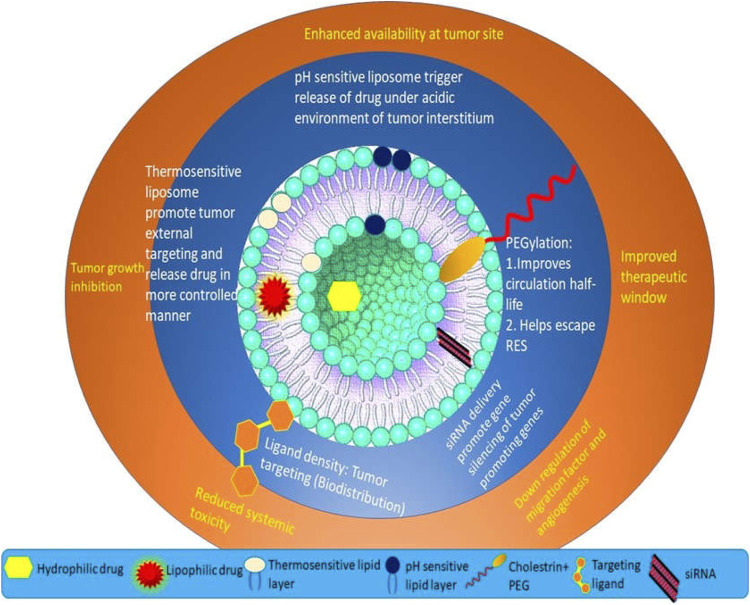
The scope of liposomes in mediating anti-cancer therapy.

The FDA has approved several liposome based anti-cancer preparations that have shown promising results even in the last stage of clinical trials. Thus, not only the safety but also the therapeutic profile of anti-cancer agents could be improved. Ever since the development of the first liposomal product, Doxil® (Barenholz, 2012), many more anticancer formulations have been successfully developed, including Depocyt® (Murry and Blaney, 2000), Mepact® (Alphandéry et al., 2015), Onivyde^TM^ (Drummond et al., 2006), Myocet® (Leonard et al., 2009), DaunoXome® (Forssen, 1997), and Marqibo® (Webb et al., 1995). The attributes of liposomes could also be modified depending on the kind of lipids used. In a current study, liposomes composed of porphyrin-phospholipids showed the simultaneous release of cargos upon NIR irradiation (Carter et al., 2014). The composed liposome 1,2-dioleoyl-sn-glycero-3-phospho ethanolamine (DOPE) has shown pH-dependent release, with maximum release at low pH due to the structural transformation of DOPE to hexagonal form, endorsing the destabilization of the bilayer (Soares et al., 2011). Nano-preparation using liposomes also has the potential to deliver poorly water-soluble agents even without adding a surfactant, which can be used to cause toxicity. With dedicated research, many novel liposomes such as pH and thermosensitive, long-circulating, and environmental sensitive liposomes have been developed (Figure 1). Two preparations, using Paclitaxel loaded liposomes (EndoTAG-1, Medigene AG and LEP-ETU, Neopharm, Inc.) reached Phase II clinical trial, which disregarded the use of Cremophore/Kolliphore adjuvant, whose usage causes severe anaphylactic shock. Thus, the maximum tolerated dose of paclitaxel was elevated (Koudelka and Turánek, 2012). A study performed by Petersen et al. showed that 11 published results prolonged the survival time in 11 tumor-bearing mice models treated with doxorubicin liposomal preparation as compared to plain drug. A meta-analysis for screening progression free survival (PFS) and overall survival (OS) using eight clinical studies was performed, comparing the efficacy of anthracycline-based liposomal preparations and conventional anthracycline preparation. No evidence was found claiming PFS and OS in patients. However, the safety profile of liposomal preparation improved. Particularly, the liposomes could not cross the endothelial lining of the blood vessel of the heart, thereby regulating the pronounced cardio-toxicity as compared to the conventional drug (Gh et al., 2016). Such, an early delivery system depended on the passive release process, which ascertains the stimulated release other than the passive release at a specific cancer cell. Due to lack of specificity, liposome’s talent remains vague by not delivering the required amount to the desired area. To overcome such shortcomings, targeted drug delivery is being introduced in the menu of research. Steady release in pH mimicking tumor environment and remote triggering such as using ultrasonic waves and photo-activating substances, modify the drug release profile. The cell selectivity of liposomes by cancer cells could be improved by the surface functionalization of liposomes. This target mediates therapy and could reduce the toxicity, improve the site-specific drug accumulation and internalization along with promoting endosomal escape (Choudhury et al., 2018; Kesharwani et al., 2018).

Incipient tumor cells acquire several biological functions and undergo metabolic changes in the journey of evolution, which permit the cells to become more tumorigenic and eventually invasive (Hu et al., 2015; Kesharwani and Iyer, 2015; Wang et al., 2020). Cancer cells over-express various surface receptors which help them to grow and proliferate (Kesharwani et al.,2011; Kesharwani et al., 2014a; Kesharwani et al.,2014b; Kesharwani et al., 2015a; Kesharwani and Iyer, 2015; Amjad et al., 2017). This accelerates their survival, challenging the chemotherapeutic activity to work against them. Integrins are also among these kinds of receptors, generally and transmembrane receptors have been implicated as playing a prime role in regulating the metabolic process by advocating cellular growth at ground level after their attachment with the ECM. Furthermore, after adhesion with ECM, integrins could regulate traction for invasion and cell migration. To summarize, integrins stimulate the survival, proliferation, and metastasis of tumorigenic cells (Yee et al., 2008; Böger et al., 2014; Duro-Castano et al., 2017). Specifically, they stimulate overexpression of αvβ3 integrin receptor, which has been allied with various types of cancer including glioblastoma, colon, melanoma, breast, pancreatic, prostate ovarian, and cervical cancer. An interesting study by Pierschbacher and Ruoslahti in 1984, described the Arginine-glycine-Aspartate (RGD) cell adhesion sequence, an extremely preserved minimal integrin sequence in fibronectin (Ruoslahti, 1996). This sequence is the smallest that can bind with the αvβ3 integrin receptor overly expressed on the tumor environment. The expression of integrins thus requires the design of agents that can bind with them using RGD as a targeting ligand. Liposomal preparations based on the delivery of chemotherapeutic agents on an integrin over-expressed site could offer multiple ranges of benefits.

This review sheds light on integrin-targeted cancer cells using liposomal preparations functionalized by RGD peptide motifs for attaining the targeted effect. Such a targeted approach is reported as a cell penetration facilitator engulfing theranostic (therapeutic + diagnostic) agents into targeted spots. It focuses attention on the role of integrin in cancer prognosis and also explains its interaction with RGD peptides. This review also highlights the RGD anchored liposomal delivery for various cancers, to compile all available current research.

## The Biology of Integrin in Cancer Prognosis

Integrins are cell surface receptors that are a prominent component of extracellular matrix (ECM), from the generation of 24 transmembrane heterodimers. To date, 18α and 8β subunits have been described on the human genome as having different affinities and specificities towards different ligands (Hynes, 2002; Humphries et al., 2006). For instance, αvβ3 integrin binds an eclectic range of ECM molecules, together with fibrinogen, vitronectin, von Willebrand factor along with proteolyzed forms laminin and collagen, while α5β1 integrin selectively binds fibronectin (van der Flier and Sonnenberg, 2001) The heterodimers of integrin are transported to the Golgi apparatus from the endoplasmic reticulum, which after modification relocated to the cell in an inactive form (De Franceschi et al., 2015). The amino acid terminals of α and β integrins bind together by non-covalent attraction, subsequently giving rise to integrin αβ heterodimers (Seguin et al., 2015).

### The Role of Integrin in Cancer Cell Survival

**FIGURE 2:**
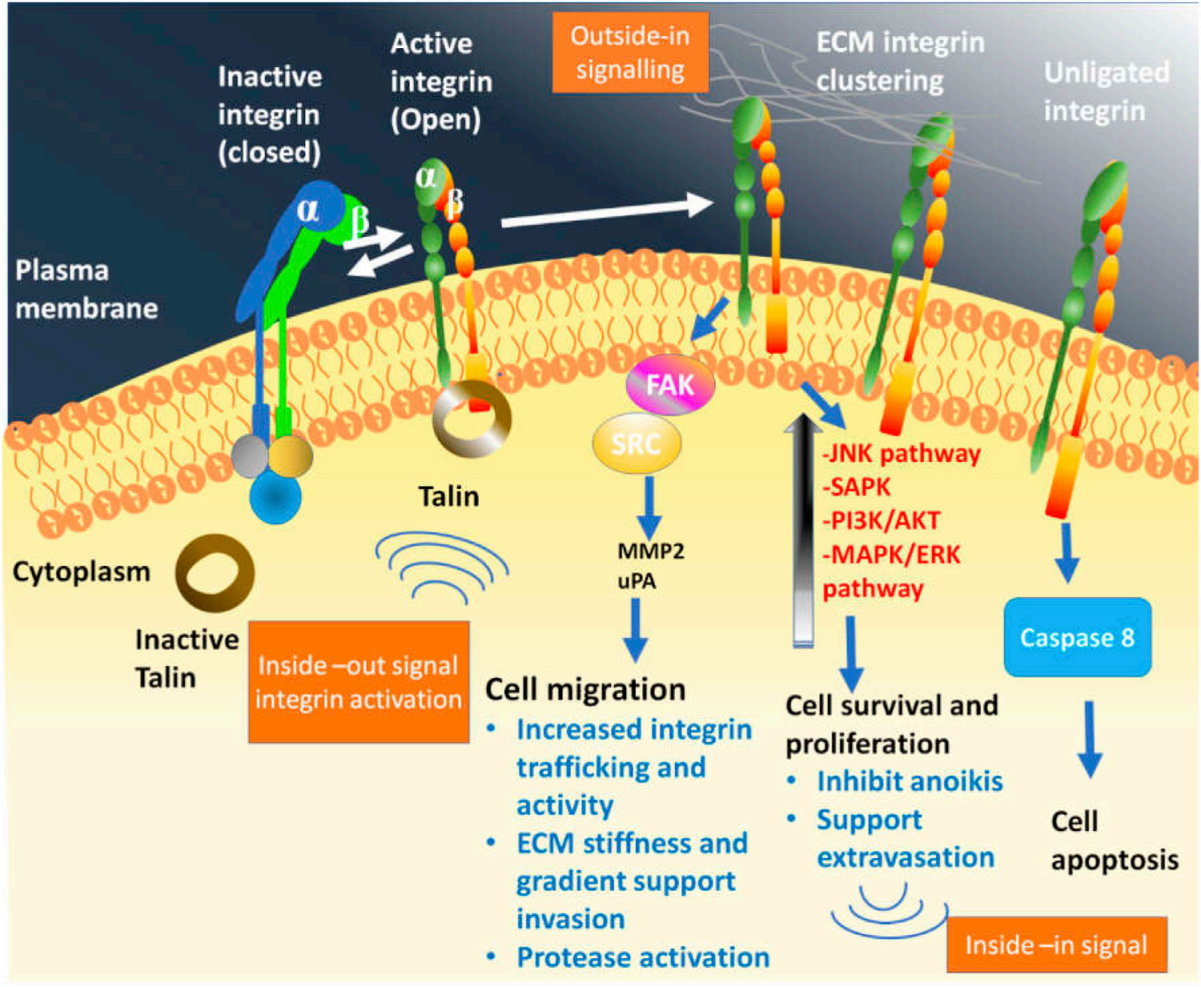
Bidirectional signalling by integrin family which is in virtue to confirmational state demonstrate its binding affinity towards extracellular matrix (ECM) and related protein. The bend or closed integrin epitomize the inactive form having low affinity towards ECM, while the straight-extended and upright integrin corresponds to the active form that show high affinity towards ECM, thereby promoting down-stream signalling eliciting cellular response following the ligand attachment. Following the integrin-ligand engagement also called as adhesion, and their clustering on plasma membrane, assembles the multimeric complexes to promote outside-in or downstream signalling. In consequence, phosphorylation of focal adhesion kinase (FAK) initiates, which further activate the steroid receptor coactivator or SRC. In association to this, other metabolic pathway-PI3K/AKT, MAPK/RAS also get activated to produce signal nodes. Talin bind to the β-integrin subunit tail which triggers the open structure. Moreover, integrin dependent signalling compounds and activated integrin in association with receptor tyrosine kinase and ECM encourages the “inside-in” signalling.

Integrin directly interacts with the component of the extracellular matrix (ECM) to arbitrate cell adhesion, which is an essential step to carry out several metabolic processes that promote cell growth and viability. Additionally, they directly regulate the invasion and migration of tumor cells after interaction with ECM components, providing a platform for cellular motility and intravasation (Guo and Giancotti, 2004). This property of integrins in tumor cells (relocation and invasion) plays important role in cancer evolution and has recently been reported elsewhere (Aoudjit and Vuori, 2001; SK and DD, 2006). The role of integrins depends on the environmental cues that could either elevate survival or initiate apoptosis (Figure 2). The normal physiology of the human body works to maintain a balance in the functioning of all processes. While on the one hand, the unlighted form of integrin encourages the pro-apoptotic cascade, simultaneously the ligated form of integrin transmits bidirectional survival signals. Such homeostasis upholds the integrity of various cells and organs by averting cell survival. (Askari et al., 2009; Legate et al., 2009; Hamidi and Ivaska, 2018; Cooper and Giancotti, 2019). The activation and affinity of integrin towards ECM increase after recruiting the integrin tail region with adaptor protein talin causes a confirmational change. Moreover, as a consequence of Integrin–ECM interactions at the integrin’s horizon the absorption of focal adhesion is promoted. Ligated integrins in the case of ligated integrins promote the activation of metabolic pathways such as MAPK, PI3K-AKT, augmented nuclear factor-κB (NF-κB), reduced p53 expression, and amplified expression of B-cell lymphoma-2 (BCL-2) and CFLAR gene. The crosstalk between the integrins and the growth factor receptors also preferentially stimulates Raf, mediating cell survival and proliferation, which in turn encourages Ser 338-9 related Raf phosphorylation, inhibiting the risk of cellular death through the intrinsic pathway. While, phosphorylation of Tyr340-1 prevents cell death through the extrinsic pathway (Legate et al., 2006; Landen et al., 2008). The unligated integrins cleaved caspase-8, prompting integrin-mediated death (Stupack et al., 2006). Cell death gets initiated after complete loss of adhesion through a process termed anoikis which proceeds either via intrinsic or extrinsic pathways (De Franceschi et al., 2015).

### RGD–A Designed Ligand of Integrin

The sequence of RGD was the smallest cell adhesion sequence discovered over 3 decades ago. The recognition sites of RGD were also obtained in other ECM proteins (Lawler et al., 1988) such as vitronectin, osteopontin (Horton et al., 1995), bone sialoprotein, fibrinogen, fibronectin, and laminin (Grant et al., 1989), whose receptors were recognized in the integrin family. The recognition by the integrin receptor depends upon the structural conformation of peptide (RGD). Integrin modulated cancer cell surfaces offer a horizon for such peptides to be eventually identified and offer a target binding site (Ali et al., 2017; Wu et al., 2017). A modulation of nanocarriers with RGD binding motif demonstrated high affinity towards cancer cells overexpressing integrin and also offered potential efficiency to conquer tumor progression via inhibition of integrin facilitated functions (Zhang et al., 2011; Ahmad et al., 2021).

### The Fate of RGD in the House of Integrin

The role of RGD is exploited in cellular adhesion, spreading, focal-adhesion, and actin-skeleton formation with integrins that are required for the transmission of signals that regulate the cell cycle and its behavior (Hwang et al., 2007; Bellis, 2011). The binding of RGD-nanoparticle to ECM ligand forces integrin to trigger “outside-in” signaling to enhance the downstream signaling for arresting the cell cycle and promotion. In synergy, the ligand-bounded integrins get internalized into the cells via clathrin-mediated endocytosis for focal adhesion turnover and then transported to late lysosomes or endosomes (Jain et al., 2012; Onodera et al., 2013; Nieberler et al., 2017; Kesharwani et al., 2018).

Few integrins separate from the binding ligands in response to the acidic atmosphere of late endosome/lysosome, and the free integrin returns to the plasma membrane (Ezratty et al., 2009). This framework of recycling integrin towards plasma membrane eventually results in the progression of cancer due to upregulation of neoplastic cells. Such processes (endocytosis or integrin return) promote the binding, internalization, and accumulation of integrin-targeted nanoparticles (Danhier et al., 2012; Dozynkiewicz et al., 2012). As observed in a study by Li et al., modified RGD conjugated nanoparticles facilitate *in vivo* anti-tumor efficacy (Li et al., 2014). A highly potent αvβ3 ligand, i.e., cyclic RGD-tyrosine-lysine peptide (cRGDyk) modified liposome encapsulating cisplatin elevated therapeutic efficacy against bone metastasis in congruency with pain relief, tumor regression, and improving overall survival improvement (Wang et al., 2014). The selectivity of RGD also depends on its specificity to bind to integrin. For instance, the appointment of integrins β3 is crucial for the cell to cell or cell to matrix interactions, thus connecting a wide network of biological processes (Bialkowska et al., 2000; Arias-Salgado et al., 2003).

In contrast to the αvβ3 integrin, which is generally associated with almost every cancer type, other αIIbβ3 integrins are encountered on the cell surface and perinuclear region of prostate neoplastic cells (Bello et al., 2000; Brüning et al., 2001; Rolli et al., 2003; Müller and Bosserhoff, 2008). However, αvβ3 and various integrin are expressed in normal cells as well but to a low extent. For example, cyclic-RGD peptides or cRGD targeted various integrin including αvβ3 αvβ8, αvβ6, and αvβ5, which were expressed on healthy tissues also, and ultimately produced toxicity in normal cells. Another major issue encountered was that RGD peptide could only recognize αvβ3, but integrin αIIbβ3 was not recognized by the RGD that were present in the perinuclear region of prostate tumor. This accelerated the demand for such modified peptides or specific ligands being taken up by integrins β3 for improving anticancer treatment and minimizing adverse effects. A molecular docking study identified an RGD modified peptide- Arginine-Tryptophan-(D-Arginine)-Asparagine-Arginine named β3Integ that showed high affinity towards integrins β3. In comparison to cRGDyK modified liposomes, β3Integ exhibited 3.3-fold anti-cancer effect. Moreover, no cardiomyocyte toxicity or weight variation change was observed after treatment with β3Integ grafted preparation (Zhang L. et al., 2019). In another approach, glutamic oligopeptides-RGD peptide (GLU6-RGD) derivative modified liposome displayed outstanding *in vivo* targeting activity in metastatic bone malignancy with high hydroxyapatite (HAP) binding efficiency, improved cytotoxicity and enhanced stability (Zhao et al., 2019). Such dual targeted effects elevate the anti-cancer efficacy of targeted nano molecules.

## RGD Engineered Liposomes as Arsenal Against Carcinoma

With many attractive properties, liposomes are a well-recognized nanocarrier in the biomedical industry. However, liposome is not new to the industry. Since the success of Doxil (Doxorubicin loaded liposome) in 1995, liposome made its way from the lab to the clinic (Seynhaeve et al., 2013). With the approval of the FDA, several liposomal preparations were promoted from the bench to bedside proving their efficacy in the last stage of the clinical trial. The significant approach of liposomal therapy is sufficient drug loading, congruous size, and very importantly, steric stabilization, which is necessary for improving circulation time (Allen and Cullis, 2013). Parameters such as controlled release with maximum efficacy of liposomal therapy are essential, which in the case of early liposome is mediated by passive release pattern. Here, due to the leaky vasculature of the tumor, the liposome loaded active anti-cancer drug elicits its action (Andresen et al., 2005; Kesharwani et al., 2015b; Luong et al., 2016; Srivastava et al., 2021), but affects normal cells as well. With insufficient release pattern, SPI-077 liposomes, despite getting internalized in cancer interstitium was not able to meet the efficacy demand (Bandak et al., 1999). Therefore, to meet the demand and provide the clinic with a candidate with a wide therapeutic window, liposomes could be grafted with ligands (Ruoslahti, 2012). By facilitating more uptake by tumor cells, serving in better internalization, and honing the anti-tumor effect, targeted liposomes could follow various pathways to achieve such objectives. Endocytic pathways such as flotillin (<100 nm), caveolae (<80 nm), and clathrin (<300 nm) mediated pathways are employed by liposomes for delivery of ligand mediated selected uptake (He et al., 2010; Bae and Park, 2011). As discussed previously, integrin is over-expressed in numerous cancers, which could be well recognized by RGD peptide. Thus, utilizing the advantage of RGD as ligands and properties of liposomes, multiple researchers have examined its effect on tumor growth. The section below illustrates examples of RGD modified liposomes with efficacy against various metastatic and non-metastatic cancers (Table 1).

**TABLE 1:** Representation of Integrin targeted Liposomal preparation modified with RGD peptide.

Composition of liposomal preparation	Particle diameter and zeta potential of targeted preparation	Anti-cancer molecule/gene therapy	Type of animal model	Type of cancer	Outcome of the study	References
Phosphatidylethanolamine, Cholesterol, Cholesteryl hemisuccinate (CHEMS)	129.61 ± 3.2 nm and −26.38 mV	Docetaxel (DTX)	Female Balb/c nude mice	Breast cancer	Under same concentration, RGD based Ph sensitive liposome showed enhanced cytotoxicity than plain DTX and non-targeted liposome due to the tumor homing effect	Chang et al. (2015)
Phosphatidylethanolamine, cholesterol, PEG 2000, Linoleic acid	146.4 ± 4.4 nm and −31.82 mV	DTX	Female Balb/c nude mice	Breast cancer	Dual targeting and PEG modified liposome enhanced the uptake while prolonging the circulation time	Zuo et al. (2016)
Cholesterol, phospholipid, Fructose, RGD	113.6 ± 2.1 nm and 4.20 ± 0.17	Paclitaxel (PTX)	Kunming and Balb/c mice	Triple negative breast cancer (TNBC)	The targeted preparation showed 2.62 times higher accumulation than non-targeted liposome	Pu et al. (2019)
Mal-PEG-DSPE, RGD	60.85 nm and −42.3 mV	Diacedic norcantharidin (NCTD)	Nude mice	TNBC	The tumor growth and metastasis reduced after treatment with RGD modified preparation	Li et al. (2019)
1,2-dioleoyl-3-trimethylammonium-propane (DOTAP), 1,2-distearoylphosphatidylethanolamine-methoxy-polyethylene glycol (DSPE-PEG2000), cholesterol, cRGD	120.21 ± 5 nm and 11 ± 1.1 mV	miR-34a	—	TNBC	The targeted liposomes possibly improved the therapeutic efficiency of mRNAs in breast tumor cells and CSCs	Vakhshiteh et al. (2020)
DSPE-PEG2000-R8-RGD, SPC, Cholesterol, DSPE-PEG2000	105.9 ± 0.7 nm and −4.95 ± 0.59	PTX	Balb/c	Glioma	The tandem R8-RGD peptide improved transportation of drugs across BBB, enhanced penetrability and tumor targeting	Liu et al. (2014)
Dipalmitoyl phosphatidylcholine (DPPC), cholesterol, TPGS, RGD	182.3 ± 7.5 nm and 1.10 ± 0.25	Docetaxel	Charles Foster Rats	Glioma	RGD-TPGS decorated theranostic liposomes were 6 fold more effective than plain DTX	Singh et al. (2016)
Mal-PEG3400-DSPE, HSPC (hydrogenated soy phosphatidylcholine) and mPEG2000-DSPE, cholesterol	115.17 ± 1.01 nm, —	Doxorubicin	BALB/c	Glioma	The multifunctional targeted drug delivery system improved the uptake and anti-glioma activity	(Z et al., 2017)
Methoxypolyetheleneglycol (Mw 2000)-distearylphosphatidylethanolamine (DSPE-PEG), SPC, Cholestrol, RGDm	211 nm, —	Doxorubicin	C57BL mice	Melanoma	RGDm modified preparation at dose 5 mg/kg of DOX prolonged the survival time manyfold	Xiong et al. (2005a)
Methoxypolyetheleneglycol (Mw 2000)-distearylphosphatidylethanolamine (DSPE-PEG), SPC, Cholestrol, RGD	—	Doxorubicin	C57BL/6 mice	Melanoma	The circulation time and tumor accumulation was demonstrated after treatment with targeted preparation	Xiong et al. (2005b)
Methoxypolyethelene glycol (Mw = 2000)–distearyl phosphatidylethanolamine (DSPE PEG), Cholestero, egg phosphatidylcholine (EPC), RGD	90.54 ± 0.34, 1.01 ± 0.65	Combretastatin A-4 and DOX	C57BL/6	Melanoma	Co-encapsulation of vascular disrupting and anti-cancer agents revealed excellent results for tumor therapy	Zhang et al. (2010)
DSPE PEG 2000, egg phosphatidylcholine, cholesterol, monomeric cRGD peptide (mo-RGD), dimeric cRGD peptide (di-RGD) and flexible dimeric RGD peptide (di-P-RGD)	100 nm, —	—	C57BL/6	Melanoma	Delivery system designed based on concern over receptor clustering improved the binding potential of RGD	Guo et al. (2014)
DSPE, Cholesterol. TH peptide with a terminal cysteine [AGYLLGHINLHHLAHL (Aib) HHIL-Cys], TR peptide with a terminal cysteine [c (RGDfK)-AGYLLGHINLHHLAHL (Aib)HHIL-Cys]	126.4 ± 0.566 nm and −4.56 ± 0.48 mV	PTX	C57BL/6	Melanoma	The TR- liposome enhanced the cellular internalization and improved the survival rate	Shi et al. (2015)
Hydrogenated soya phosphatidylcholine (HSPC), Cholesterol, DSPE-mPEG (2000), RGD	87.9 ± 3.9 nm, in between −19 and −21 mV	Bufalin	—	Lung cancer	The targeted therapy improved the anti-proliferation activity by prolonging circulation time and improving cellular internalization	ZhangY. et al. (2019)
Cholesterol, Dipalmitoyl-sn-glycero-3-phosphocholine (DPPC), dioleoyl-sn-glycero-3-phosphoethanolamine (DOPE), DSPE-128 mPEG 2000), cRGD	125.06 ± 4.90 and 11.78 ± 0.21 mV	siRNA	Swiss albino wistar rats	Lung cancer	The liposomal preparation with siRNA inhibited the viability of A549 cells	Khatri et al. (2014)
HSPC, mPEG2000-DSPE, cholesterol and α-tocopherol	115.9 ± 2.4, —	DOX	BALB/c	Colon cancer	RGD peptide with intermediate hydrophilicity made headway to control tumor growth and improve the survival time	Amin et al. (2013)
Egg phosphatidylcholine, DSPE-PEG2000, (DOTAP) chloride, CaCO_3_	Between 115 and 120 nm, zeta potential ranged between 20 and 25 mV	PTX + Protein (BSA)	Male ddY mice and BALB/c mice	Colon cancer	pH sensitive release of drug and targeting of nanoparticle synchronized the bio-distribution leading to significant anti-tumor activity	Peng et al. (2019)
DSPE-PEG2000, Cholesterol, RGD, TAT-Cysteine peptide	89.41 ± 0.80 and 1.18 ± 0.92	—	HepG2 xenograft nude mice (specie not explained)	Liver cancer	The synergic effect shown by RGD and TAT modified liposomes enhanced the lysosomal escape, clustering the nanocarrier in the cytoplasm	Mei et al. (2014)
DSPE-PEG, Chlorodimethyloctadecylsilane, distearoyl phosphatidylcholine (DSPC), RGD	152 nm, 20 mV	Arsenic trioxide	H22 tumor xenograft mice	Liver cancer	The nanocarrier with controlled release and targeted therapy modified the anti-cancer potency of arsenic trioxide	Fei et al. (2017)

### Breast Cancer

Accounting for more than one per 10 new cases, breast cancer ranks top in the most commonly diagnosed cancers among women (Alkabban and Ferguson, 2021). Its therapy involves using drugs that block cancer growth by interfering with respective molecules that hinder proliferation and survival. The cells may overexpress specific receptors, which upon activation promote downstream signaling, and in consequence result in the countenance of certain genes for cancer cell growth, proliferation, migration, survival, angiogenesis, and promotion of other vital cell cycle pathways (Yarden and Sliwkowski, 2001; Murphy and Dickler, 2016). A thorough study of over-expressed receptors could provide a platform for target-based therapy.

Over-expression of integrin in breast cancer cells mediates cell growth after binding with integrin. Hence numerous molecules are being developed towards integrin overexpressed cells, which help with tumor apoptosis and cell death. The liposomes derived from model biological membranes manifest the high potential of gene and drug delivery, molecular imaging, and employ biological cell membranes that mimic the human microenvironment. To achieve extra therapeutic benefits, targeting numerous components such as proteins, antibodies, peptides, and small molecules could reduce the off-target effect of naked nanoparticles (Torchilin, 2005; Ying et al., 2010; Manjappa et al., 2011; Muthu et al., 2011; Wang et al., 2012). The pH-based drug delivery system also earmarks the tumor by releasing cargo in the acidic environment of the tumor (6.5–6.7), while remaining intact at physiological pH. This type of smart, target-based approach guarantees reduced toxicity and elevated therapeutic effect.

To utilize extra-ordinary benefits such as controlled properties and high uptake potential, Chang et al., developed RGD anchored Cholesteryl hemisuccinate (CHEMS) based pH responsive liposome loaded with docetaxel (DTX). CHEMS are protonic lipid having ester derivative at 3-hydroxyl group, utilized in constructing pH responsive drug delivery system. The prepared DTX loaded liposomes (D-LP) and RGD anchored D-LP (RGD-LP) displayed pH dependent size variation. The size of D-LP and RGD-LP increased from 136.4 to 129.4 nm to 961.4 and 685.2 nm with a shift in pH from 7.4 to 4. This is due to the presence CHEMS, which significantly promoted drug release at definite pKa. The targeted preparation showed maximum intensity around the nuclei which indicated selective uptake of ligand bound liposomes in MCF-7 cells bearing mouse model (Chang et al., 2015). Hence, RGD based liposomal therapy could be a promising opportunity for multifunctional drug delivery systems.

In a similar study by another research group, pH sensitive DTX loaded liposomes decorated with PEG linked RGD peptide as targeting ligand have been investigated using a different polymer, Linoleic acid (LA). LA is anionic at physiological pH, however, protonation of unbounded carboxylic acid at the acidic environment (tumor site) alters the confirmational structure, causing destabilization of liposome leading to its collapse or dissociation. As compared with the non-targeted liposome and DTX (Duopafei^®^), RGD-decorated liposome showed larger AUC, extended mean half-life, and residual time (MRT) along with a slower clearance rate *in vivo*. The superior performance is ascertained due to the presence of a PEG chain that connected RGD and LA. The linked and unlinked PEG with RGD, swing on the liposomal surface, thereby prolonging the circulation time. The plain liposomes did not exhibit any cytotoxicity in MCF-seven cells, with more than 89% surviving cells, indicating the safety of liposomes in a biological membrane. The IC50 value of RGD grafted liposomes, non-targeted liposome, and Duopafei^®^) after 72 h was 1.05 ± 0.04, 1.50 ± 0.13, and 1.84 ± 0.10 μg/ml, respectively. Moreover, the smallest mass of tumor was observed in mice treated with RGD bound liposome, which was attributed to good tumor-inhibiting potential due to enhanced uptake by tumor cells. Overall, the preparation represents a new strategy for drug delivery systems with a dual targeting effect to suppress tumor progression (Zuo et al., 2016).

Triple negative breast cancer or TNBC accounts for nearly 20% of all breast carcinoma and is characterized as the most aggressive form of cancer. Currently, for the treatment of TNBC, chemotherapy, radiotherapy, and surgery remain options for the treatment. However, due to meager prognosis and high mortality, the dilemma of treatment should be alleviated urgently (Chang-Qing et al., 2020; Medina et al., 2020). To acquire the best therapeutic response and clinical output, one must understand the normal physiology of the tumor environment. Cancer cells are depleted of oxygen and glucose, which steal nutrients from the normal cells. In contrast to normal cells, tumor cells are depicted by a sharp rise of glucose uptake in an oxygen rich environment, known as the Warburg effect (Gowrishankar et al., 2011). Thus, the Warburg effect, together with proliferation consumes more glucose, making a low-glucose microenvironment in tumor cells.

Breast cancer cells consume fructose 8-10 times higher than the normal cells. Selective from different kinds of hexose transporters, solitary GLUT2 and GLUT5 was capable of carrying fructose (FRU), among which GLUT5 wons the race (Zamora-Leon et al., 1996; BJ et al., 2009; Sheng et al., 2009).

**FIGURE 3:**
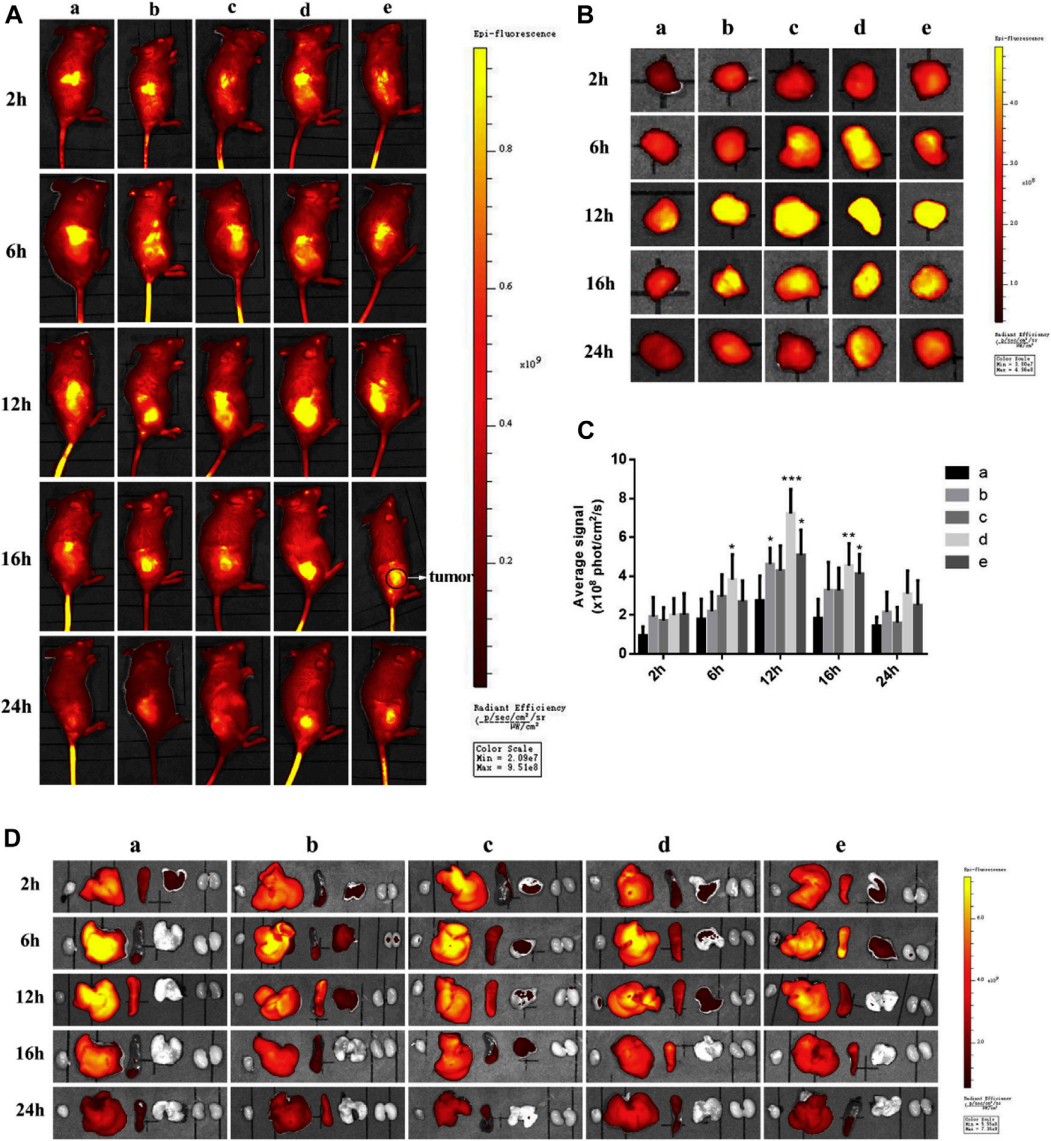
*In vivo* **(A)** and *ex-vivo* images **(B)** 4T1 bearing mice treated with **(i)** DID-Lip **(ii)** DID-FRU-Lip **(iii)** Did-RGD-Lip **(iv)** Did-RGD/FRU/Lip and **(v)** Did-RGD-FRU-Lip. Among the modified liposome **(iv)** showed strongest fluorescent intensity due to targeting effect. (https://doi.org/10.1016/j.ejmech.2019.111720).

The environment of the tumor along with sufficient information regarding over-expression of receptors have led to the design of targeted drug delivery systems with promising essential candidates against cancer. To improve targetability, Pu and team reported two novel types dual targeting liposomes loaded with Paclitaxel (PTX). One is obtained through the physical mixture of liposome with RGD (targeting αvβ3 integrin over-expressed on TNBC cells) and fructose (to target cells showing Warburg effect) forming RGD-FRU-Lip, another is the covalently linked liposomes that formed Y shaped ligand with RGD and FRU (RGD/FRU/Lip). The results manifested that RGD/FRU/Lip was stable and safe to use, showing hemolysis, which is regarded safe. As compared to free liposome (lip), RGD-lip, FRU-Lip, and RGD-FRU-Lip, RGD/FRU/Lip showed significant cytotoxicity, while free PTX exhibited the least cell viability due to the passive diffusion. Moreover, the upregulation of RGD/FRU/Lip was also significantly enhanced than other preparations, as revealed by confocal microscopy. The result of confocal microscopy showed cytoplasmic localization of liposomes that rarely entered the nucleus facilitated by the release of drugs after phospholipid destruction. The *in vivo* images 4T1 bearing mice showed maximum targetability 12 h post-treatment with DID-RGD/FRU/Lip. Solely modified liposomes such as DID-RGD-Lip and Did-FRU-Lip also showed comparable results to unmodified liposomes. Even after 24 h, the modified liposomes present in the cancer cells were significantly greater compared to the remaining groups (Figure 3). Thus, researchers studied the environment of the tumor and designed a therapeutic regimen that reduced the undesired effect on normal cells while improving targeting ability (Pu et al., 2019).

For decades the fight against cancer has sought to realize β-catechin as a potential mediator of the Wnt pathway as it plays an important role in homeostasis and expansion of adult tissue *via* mediating the embryonic and stem cell functions. Particularly in TNBC, this pathway is superiorly activated, contributing towards initiation and metastasis. Thus, to rationalize, Li and team developed Diacedic Norcantharidin (NCTD), an anti-cancer-loaded RGD-coated lipid polymer hybrid (RGD/LPH). They realized that TNBC cells expressed integrin α5 (ITGA5) expression to a far greater extent than normal cells. The targeted formulation showed promising results that enhanced the circulation time and accumulation of NCTD in mice with orthotopic mammary TNBC tumor and lung cancer cells showing metastasis, which offered a platform for a targeted approach in attenuating TNBC tumor. *Via* downregulating β-catechin, the tumor growth and metastasis also reduced in RGD/LPH treated tumor bearing mice as compared to LPH/NCTD and free NCTD, inflicting the tumor homing effect *via* binding with integrin (Li et al., 2019). Apart from the delivery of drug based anti-cancer substances, gene therapy also offers a great therapeutic window in cancer therapy. Micro RNA or miRNA are demonstrated in regulating gene expression in cancer and its related conditions (Huang et al., 2013; Mirzazadeh Tekie et al., 2015; Assali et al., 2018). It has been revealed that miR-34a influences cell arrest and apoptosis, however, the major challenge is stability. Nucleases degraded the nucleic acid, thereby reaching the target site in an inactive form. Thus, to deliver miR-34a successfully to the cancer site, Vakhshiteh and team focussed on the ability of liposomes to protect and deliver the genetic agent. To achieve targeted delivery the miR-34a loaded liposomes were PEGylated (to improve the circulation time and protect liposomes from interaction with serum protein) and cRGD to target TNBC and circulating stem cells (CSCs) over-expressing integrin receptors. The targeted liposomal preparation resulted in higher cellular uptake (2X), which indicated miR-34a stability amplification and increased cellular uptake. The preparation also affects the migration of tumor cells by 33% after treatment with targeted liposome than non-targeted liposome, which reduced the cellular migration by 15%. Similarly, invasion of cancerous cells also reduced significantly by 17 and 8%, after being treated with targeted and unligated liposomes. This is due to the higher uptake of cRGD decorated liposomes, which effectively augmented the miR-34a accumulation, thus, substantially curbing invasiveness and malignancy (Vakhshiteh et al., 2020).

### Glioma

Gliomas are a subtype of Central Nervous System (CNS) neoplasm, that originates from the neuroglia such as oligodendrocytes and ependymal cells of the brain. Among all brain cancer, glioma accounts for nearly 30% and approximately 80% among primary malignant brain cancers. However, the aetiology is still unknown (Mishra and Kesharwani, 2016; Reifenberger et al., 2017; Malta et al., 2018; Molinaro et al., 2019; Lin et al., 2020). Today, the standard treatment is surgery followed by chemotherapy and radiotherapy. Limited penetrance of the therapeutic agent and resistance due to blood brain barrier (BBB) and blood brain tumor barrier (BBTB), diminishes the overall 5-years survival rate. Liposomes have gained attention in the therapy of glioma due to their hydrophobic nature, facilitating the diffusion of hydrophilic or lipophilic drugs across the BBTB and BBB (Ruan et al., 2021), and thus accumulating in brain cancer cells. Non-targeted liposomes, however, pose a hurdle as they raise the toxicity by also liberating drugs in normal cells. This explains the value of targeted preparation that avoids off-target drug release (Shi et al., 2008).

In a study by Liu and co-workers, the PTX loaded liposome was modified with cyclic RGD conjugated with another cell penetrating peptide (R_8_), to obtain R_8_/cRGD liposomal structure. In contrast to linear RGD, cyclic RGD shows 1,000 times greater targeting ability. On C6 cells, R_8_-RGD-Liposome and R_8_-Liposome showed a strong anti-proliferation effect than free PTX due to higher cellular uptake. However, blank liposome exhibited no toxicity, which illustrates its safety. Moreover, R_8_-RGD-Liposome induced stronger apoptosis (38.30 ± 3.11%) as compared to other preparations. This indicates that R_8_-RGD augments the cellular accumulation of targeted preparation, releasing more drugs directly to the cytoplasm besides attaining *in vitro* inhibition. Additionally, PTX-R_8_-RGD-lip treated mice bearing glioma showed improved efficacy by elevating the survival time to 48 days, which was significantly longer than those treated with free PTX, PTX- R_8_-lip, and PTX-RGD-lip, showing survival of 32, 36, and 38 days, respectively (Liu et al., 2014). In another interesting study, Docetaxel (DTX) and quantum dot (QD) loaded liposomes coated with *d*-alpha-tocopheryl polyethylene glycol succinate (TPGS) and RGD were prepared, which shows the ROS scavenging property of TPGS. As compared to non-targeted liposomes (DTX/TPGS/Lip), non-targeted theranostic liposomes (DTX/QDs/TPGS/Lip), plain DTX, and plain QD, DTX/QDs/RGD/TPGS-Lip remarkably reduced ROS levels. The theranostic liposome shadowed integrin-receptor released the therapeutic agent via endocytosis at a specific site that additionally reduced ROS production in the brain. Moreover, the preparation was biocompatible showing no signs of toxicity as evaluated *via* histological study. Thus, it could be concluded that due to over-expression of integrin, transport of both active moiety and imaging agent, simultaneously, could be potentiated (Singh et al., 2016). A multi targeted approach has also been recently introduced in the treatment of glioma therapy. This could overcome the limitations imposed by BBB and BBTB. To examine enhanced benefits, a study recently reported that two ligands, cRGD, could recognize αvβ3 integrin and small ligand i.e., p-hydroxybenzoic acid (p-HBA), which could target BBB, and which has been connected via appropriate linker forming cRGD-p-HBA, then covalently with Mal-PEG_3400_-DSPE. The resulting Y shaped complex was then entrapped in the Doxorubicin (DOX) laden liposomes (cRGD-p-HBA-DSPE-DOX-LIP). The *in vivo* imaging demonstrated maximum fluorescence intensity at 4h, cRGD-p-HBA-DSPE-DOX-LIP was evidenced more significantly in glioma cells than the unmodified and monoligated liposomes, suggesting that the liposomes decorated with Y shaped agents could facilitate *in vivo* targeted brain delivery. The anti-cancer efficacy of Y-shaped ligand modified liposomes was demonstrated by the U87 glioma-bearing mice model. Credit goes to the synergistic effect mediated by cRGD and p-HBA for transporting targeted nanoparticles across brain barrier pervading, improving drug circulation time, and enhancing targeted delivery towards the brain (Belhadj et al., 2017). However, integrins could be recognized by RGD peptides, which also imposes certain malfunctioning. In a study, it was corroborated that cRGD modified liposomes caused IgG-mediated acute systemic anaphylactic along with anti-cRGD-liposome IgM and IgG production (Wang et al., 2019). The same research group employed a computer-assisted virtual screening method to produce a linear pentapeptide that could recognize the integrin effectively. In contrast to cRGD strengthened liposomes, RW liposomes exhibited better immune-compatibility, enhanced circulation along with vasculogenic mimicry targeting, improved angiogenesis, and therapeutic prowess that could hone the delivery of targeted liposome to integrin expressed cells (Li et al., 2020).

### Melanoma

Melanoma is the deadliest form of skin carcinoma. At an early stage, melanoma can be treated successfully with surgery alone with improved survival rates, but after metastasis, survival rates decline significantly. Therefore, the correct initial diagnosis is key for ensuring the best possible prognosis for patients. However, once melanoma turns advanced, surgery no longer remains the pivotal step of treatment (Tsatmali et al., 2002; Viros et al., 2014; Madheswaran et al., 2017; Singh et al., 2020; Kaur and Kesharwani, 2021). Conventional chemotherapy obstructs the way of promising candidates reaching the benchmark, hence TDDS are emerging as the most explored option. Studies have put forth the development of target assisted delivery system of therapeutics with controlled release. In addition, revolutionary sterically stabilized liposomes (SSL) can achieve targeted benefits and increase the circulation time (Immordino et al., 2006). SSL is demonstrated in cancer tissue at higher concentrations due to their enhanced permeability and retention (EPR) effect. For example, in one study SSL with poly (ethylene glycol) (PEG) at the distal end was conjugated with RGD mimetic (considered as RGDm), which due to passive accumulating and enhanced intracellular delivery, improved therapeutic efficacy (Xiong et al., 2005a). In a similar study by the same research group, RGD modified liposomes showed enhanced intracellular delivery of doxorubicin as compared to non-ligated molecules. This could be due to the conjugation of SSL with RGD; however, the drug release pattern was similar to those with SSL-DOX. Thus, it was clinched SSL and RGD conjugate could not elevate the DOX release. Since the negative zeta potential of SSL and RGD-SSL were close enough, the electrostatic between the cell membrane and liposome contributed in part to the increased DOX uptake and release. Moreover, cellular uptake was increased due to RGD binding with integrins present on melanoma cells (Xiong et al., 2005b). Combination therapies are well known when a single drug fails to deliver enough effect. Anti-vascular therapy along with cytotoxic agents could be a blockbuster treatment option. On the one hand, early metastasis and dividing rims of mature cancer cells are sensitive to cytotoxic therapy, while on the other, more mature tumor cells and angiogenic metastasis are sensitive to anti-vascular therapy (Horsman and Siemann, 2006; Pastorino et al., 2006). To validate, Zhang and team loaded anti-vascular agent, namely combretastatin A-4 (also known as CA-4) along with doxorubicin (DOX) in RGD modified liposome, which delayed the subcutaneous tumor growth in mice and was greater than those liposomes laden with individual moiety. Finally, displaying synergistic mutual therapeutics benefit to the dual effect, CA-4 firstly disordered the vascular matrix and thereby accumulated more liposomal DOX inside the cancer site liberating its cytotoxic effect. Hence, combination therapy could be more beneficial and could be effective in cancer therapy, specifically for intractable tumor diseases (Zhang et al., 2010).

**FIGURE 4:**
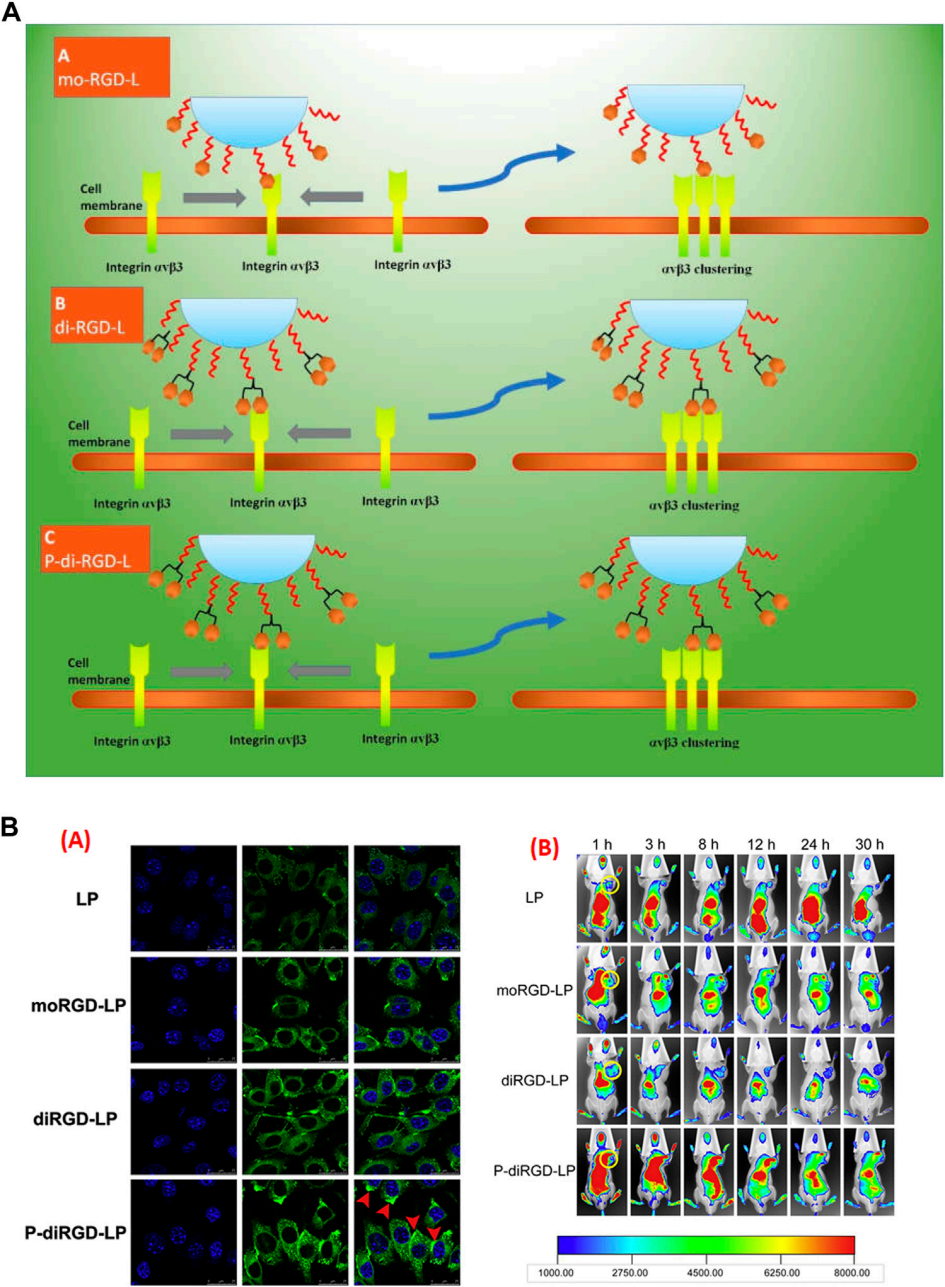
**(A)** Schematic demonstration of different RGD modified nanocarrier towards the adjacent binding site of αvβ3 integrin. In first case, mo-RGD-L molecules, the RGD moiety were too distant to bind with two αvβ3 clustering elements, in second case with di-RGD-L, the two RGD motifs were very near to each other and thus conformationally unrelated to two αvβ3 clustering elements, while P-di-RGD-L were appropriate to bind efficiently to the two integrin cluster elements. **(B) (i)** Confocal laser microscopic images (CLSM) of melanoma cells (B16) incubated at 37°C, pre-incubated with respected preparations, **(ii)** *In vivo* fluorescent images of B16-tumor bearing C57BL/6 mice treated with Liposome, mo-RGD-L, di-RGD-L and P-di-RGD-L, each loaded with DID, confirming P-di-RGD-L exhibited maximum fluorescence at 12 h, which even encountered in following hours as well http://dx.doi.org/10.1016/j.biomaterials.2014.04.031.

Over the last decade, targeted or ligated nanocarriers have been seen in an array of research for specific targeting to procure more output in melanoma therapy (Hunt and Vorburger, 2002). However, little research is concerned with the function of ligand binding on the change of receptors. In an attempt to challenge this issue, stealth liposomes were amended into monomeric RGD (mo-RGD-L), dimeric RGD (di-RGD-L), and a unique dimeric cRGD motif with a linker in-between (P-di-RGD-L). The 3-dimensional models of αvβ3 clustering proposed a receptor interval within ~ 41 to ~ 65 Å, whereas the molecular computation confirmed an RGD ligand interval of ~ 78 Å, 42 Å and 20 Å, for mo-RGD-L, P-di-RGD-L and di-RGD-L, respectively, which confirms P-di-RGD-L is the perfect match between clustered αvβ3 and special dimeric peptide conjugated liposome. *In vivo* imaging results suggested augmented uptake of P-di-RGD-L as compared to other RGD based preparation, suggesting improved endocytosis and targeting ability of liposomes for tumors, highly expressing αvβ3 integrin **(** Figure 4) (Guo et al., 2014). However, specific ligands are frequently are not enough to promote the endocytosis or penetrate solid tumor (Liu et al., 2013). Thus, team Shi aimed to modify the paclitaxel loaded liposomes-based preparation by connecting novel c (RGDfK) peptide to the C-terminus of the peptide TH through an ester bond together with TR peptide. The surface plasmon resonance study confirmed excelled binding of TR-liposome at pH 6.5 than RGD-Liposome, TH-Liposome or PEG-liposome. It was established that TR-liposomes got inside the cell through clathrin or caveolin mediated endocytosis, which was not demonstrated by non-modified liposomes. Additionally, the TH-liposomes and RGD-liposomes carrying paclitaxel did not show variable anti-cancer effects *in vivo* due to the expression of integrin on cancer cells, however, TR-liposomes carrying paclitaxel showed a synergistic anti-cancer effect, which is due more to internalization and higher specificity towards the cancer cells (Shi et al., 2015). Keeping the normal physiology of cancer in mind, one can efficiently deliver a wide network of cancer therapy. In another study, fluorescence imaging of dual targeted liposome carrying galectin-1-specific anginex (AX) and RGD peptide (AX-RGD-LIP) showed 53 ± 6% fluorescence intensity as compared to AX-LIP (43 ± 9%) and RGD-LIP (28 ± 8%). This is due to their targeting efficacy and specificity, which in turn also improved the circulation time (Kluza et al., 2012).

### Lung Carcinoma

With 235,760 new cases and 131,880 deaths worldwide, lung cancer has become the most prevalent cancer in men and the third most prevalent among women. Major risk factors of lung carcinoma are tobacco smoking, workplace exposure to asbestos or arsenic, or environmental exposure to certain pollutants, radon, and harmful gases (Bray et al., 2018; Keikha and Esfahani, 2018; Gorain et al., 2019; Board, 2021). Lung carcinomas are broadly divided into two forms, small cell lung carcinoma, and non-small cell lung cancer. The non-small-cell cancer is further divided into adenocarcinomas, squamous cell cancer, and large-cell lung neoplasm. Adenocarcinoma generally arises in non-smokers, extends towards the periphery, and multiplies in size within 161 days, while squamous cell cancers account for nearly 30% of all lung cancer. It generally originates at the center of the chest, grows slowly, and doubles in size in 88 days. In contrast, small cell lung neoplasm account for nearly 20–25% of lung carcinoma, which tends to originate in central locations, grow swiftly, and double in size in approximately 29 days. The major population diagnosed with small cell lung cancer have metastatic carcinoma (Norouzi and Hardy, 2021). However, therapies such as conventional chemotherapy could reduce the growth of a tumor, but also affects the gastric, cardiac, and neural functions of the body. Meanwhile, nano medicines also present novel ideas that could reduce such toxicity while improving the therapeutic window, but non-targeted preparation does not provide the desired benefit.

To overcome the drawbacks related to passive targeting, ligand-based nano-carriers have been under investigation. αvβ3 integrin is largely expressed on numerous tumors including, lung, breast, colon, pancreatic and hepatic. RGD is a small sequence ligand that shows high affinity towards αvβ3 integrin over-expressing cancer cells. This was proven in a study conducted by Zhang and team, who formulated PEGylated liposome (PEG-LIP) carrying bufalin, a highly toxic and short-lived anti-cancer moiety, finally functionalized with RGD peptide. As compared to plain bufalin (BF), RGD-PEG-BF-LIP significantly inhibited proliferation of A549 (lung cancer) cells showing only 10.11 ± 2.2 viability, which was much greater than PEG-BF-LIP (13.21 ± 3.5%) and pure BF (96.6 ± 3.7%). Strikingly, PEG-BF-LIP also showed significant results because liposomes were modified with DSPE-mPEG (2000) that prolonged the release of BF. The least cell viability by RGD-PEG-BF-LIP was due to the tumor homing effect as RGD peptide was up taken by cancer cells having integrin receptor on the surface (Zhang Y. et al., 2019). Due to the transformations in research, siRNA-based therapy (Butt et al., 2016; Chadar et al., 2021) has also gained significant attention among scientists. Maximum therapeutic benefit could be achieved if such molecules could be made available at the desired site. siRNA, in a specific manner, inhibits the expression of certain genes and proteins that cause tumor growth and metastasis (Kesharwani et al., 2015a; Xiong et al., 2018). However physiological barriers such as nucleases degradation, reticuloendothelial system (RES) uptake, endothelial barrier, and the high rate of elimination are among major hurdles including anionic nature and hydrophilicity, which causes effective protein knockdown and inhibits cellular entry. To overcome such barriers, Khatri et al., prepared the aggregate of siRNA and calcium phosphate through ionic interaction and entrapped it inside the liposomal core. However, due to changes in pH, temperature, and concentration, reproducibility presents another challenge. Hence, a nanocarrier with a well-defined structure could solve this issue. To achieve targeting effect, cRGD peptide was engineered to siRNA entrapped liposomes. The targeted preparation carrying siRNA exhibited 24.2 ± 3.4% gene expression in A549 cells, while bare siRNA unveiled more than 80% gene expression.

The higher the expression of a gene; the poorer its silencing ability. Higher gene expression by naked siRNA can be explained due to low transfection in the cell, while liposomal preparation has higher transfection efficiency, which is due to the structural resemblance between formulation and biological membrane. DOPE in liposomes helped in the amalgamation of cell and endosomal membrane to the liposomes, which deliver the high cytosolic siRNA availability of siRNA with a small expression of the gene. To summarize, liposomes proved an excellent carrier for the delivery of genes, which upon modification, helped in enhancing uptake to promote gene silencing (Khatri et al., 2014).

Liposomes can entrap both hydrophilic and hydrophobic drugs. They are also an important carrier for diagnostic molecules that are required for the detection of cancer growth when extensive techniques such as MRI and X-ray fail due to a lack of inherent sensitivity. In one study, RGD engineered liposomes carrying gadolinium diethylenetriamine penta-acetic acid (GD-DTPA) (RGD-GD-DTPA-LIP) and GD-DTPA-LIP were prepared. Nanoparticles with a high payload of imaging agents along with surface modification with ligand could specifically deliver nanoparticles at the tumor region. As observed by MRI imaging in A449 tumor bearing mice, the intensity within the GD-DTPA treated group reached a maximum after 1 h, which further declined rapidly. The non-targeted liposome also showed maximum intensity after 2 h, however, its surface enhancement rate (SER) also declined and returned to the baseline 6-h post-injection. The maximal SER was observed after targeted therapy after 4 h, which was highest among all groups that also maintained maximum level for 6 h. This shows the efficiency of the ligand that helped in internalization and retention inside the desired cell (Li et al., 2011). Hence, RGD anchored liposomes could not only deliver the chemotherapeutic agent directly to the anticipated site but also make the imaging agent available at cancer cells.

### Colon and Liver Carcinoma

Colon and liver cancer are the deadliest malignancies for both sexes. Environmental associations and genetic factors contribute significantly to their growth. Due to the impact of screening and proper vaccination, the new case rate has fallen in recent years (Gelband et al., 2015; Aljabali et al., 2020; Recio-Boiles and Cagir, 2021). However, much still needs to be done that could hone to hasten the decline. For colon cancer, endoscopic resection, surgical resection, and adjuvant therapy remain the available options for therapy. However, such therapy is available for stage II and above patients, while conventional therapy could be useful up to a certain extent but could not revamp the overall survival (Dawson et al., 2019). Liver cancer is one of the least successfully treated cancers but is among the most readily prevented cancer. The major intervention for liver cancer is the anti-hepatitis vaccine. Currently, there is no standard second-line chemotherapy available for patients who do not respond to sorafenib. Moreover, hepatic or liver carcinoma is considered a chemotherapy-refractory tumor and thus chemotherapy is not given in patients routinely. Among other available drugs, doxorubicin has been studied for advanced hepatic cell carcinoma, but the results remain disappointing (Burroughs et al., 2004; Yeo et al., 2005). Personalized medicine could be a better option for therapy (None et al., 2018) but before researchers directly undertake treatment, they first need to understand the demands of the biological system. Receptor over-expression on tumor cells and their targeting has been the cornerstone of this approach. Among the various vascular markers, anti-vascular targeting of integrins αvβ3, α5β1, and αvβ5 were critically associated with tumor differentiation and its metastasis. Compared to quiescent and pre-existing vessels, a higher expression of integrin in the tumor environment makes them a promising target and target-based research is making headway.

Scientists are not just exploring the ligands, they are also modifying them to improve their targetability. This is demonstrated in a study by Amin and team. They decorated PEGylated liposomes with three peptides individually. The chosen peptides were RGD-d-Tyr-Cys (RGDyC), which is least hydrophilic, RGD-d-Phe-Lys (RGDfK), and RGD-d-Phe-(N-Methyl) Lys [RGDf (N-Met) K] (moderately hydrophilic). The doxorubicin loaded PEGylated liposome with RGDf-(N-Met)-K motif showed 2.46- and 3.93-times higher accumulation in contrast to liposome bearing RGDyC and RGDfK motif. This effect was achieved due to the direct attachment of targeted nanoparticles to vasculature mediated by EPR. The controlled and prolonged release is a prerequisite for any nanoparticle, that opens the door for attachment and effective collision or extravasation. This was the reason RGDfK grafted doxorubicin loaded liposome could not achieve greater accumulation of high tumor level despite high binding ability. The targeted therapy also showed a promising anti-cancer effect *in vivo* by increasing the survival rate and suppressing the tumor growth in mice (Amin et al., 2013).

**FIGURE 5:**
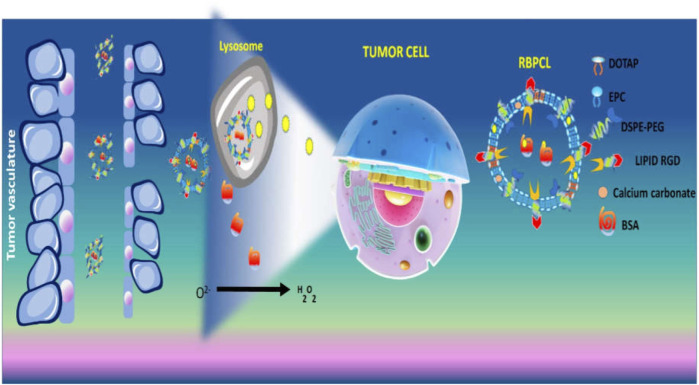
Schematic illustration of pH dependent drug release from CaCO_3_ nanoparticles containing BSA and PTX.

In another study, RGD grafted lipid-based nanoparticles carrying both bovine serum albumin (BSA) and chemotherapeutic (PTX) were prepared using calcium carbonate (CaCO_3_) forming RBPCL. The advantage of CaCO_3_ in nano-preparation is that it gets destroyed in the acidic microenvironment of the lysosome. This could increase the release of drugs in tumor interstitium. The protein-loaded nanoparticle imitated *in vivo* circulation in blood, additionally, decomposition of CaCO_3_ favourably elevated intracellular drug and protein release. Due to the RGD ligand, the uptake of nanoparticle affected the growth of tumor in colon tumor xenograft mice (Peng et al., 2019) (Figure 5). The simple PEGylation of nanoparticle can prolong circulation time by avoiding the interaction of plasma protein and quick removal by reticuloendothelial system (RES). Thus, the carrier can be accumulated at tumor site and is efficiently internalized into the cell. As every coin has two faces, PEGylation seriously affects the interaction of carriers with the cell at their arrival at tumor site (Gullotti and Yeo, 2009). To find a solution, cleavable PEG, through certain linkers on nanocarrier surface, could remove the PEG after it arrives at the tumor cell (Kale and Torchilin, 2007). Considering the “shielding” effect provided by cleavable PEG, a multistage liposomal therapy modified by RGD, cell penetrating peptide [Tat (AYGRKKRRQRRR)] and cleavable PEG has been manufactured by Mei and co-workers. After investigation of the cell trafficking of liposome, it was observed that RGD-TAT modified cleavable PEG labeled liposome (RGD-Tat-C-LIP) was majorly observed in the cytoplasm, however, few were also encountered in lysosomes. This is due to clathrin-mediated endocytosis, which suggests the mitogenic substances ultimately reached the. However, cleavable PEG and Tat co-modified liposome (Tat-C-LIP) were majorly scattered in the cytoplasmic section outside the lysosome which stipulated that both had a capability for lysosomal escape. However, PEG did not shed entirely from the surface of the liposome initially, but, after interaction with the lysosome enzyme, the PEG got detached.

**FIGURE 6:**
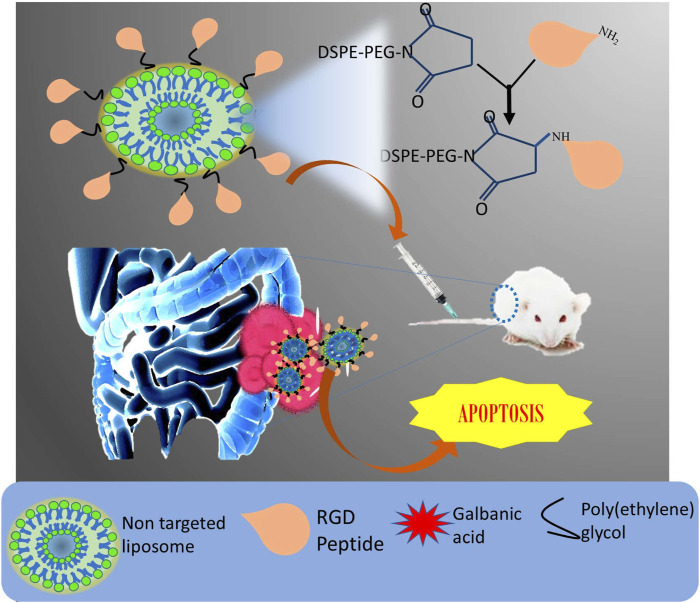
Schematic representation of preparation of RGD-decorated liposome loaded with galbanic acid against colon cancer.

Liposomes were also able to mediate successful lysosomal escape due to the membrane fusion effect of Tat. The dual targeted (Tat and RGD) liposome was strongly internalized in hepatic tumor cells in comparison to a single ligated liposome. The exposed integrin receptor bound to the RGD grafted material and sufficiently internalized the molecule inside the cell (Mei et al., 2014). The biocompatible nature of liposome makes them an important carrier for many anti-cancer drugs that show severe systemic toxicity (Bulbake et al., 2017; Lammers et al., 2008; Park, 2007). Hollow mesoporous silica nanoparticle (MSN) carrying anti-cancer agent-arsenic trioxide was coated with biocompatible liposome by Fei et al., to address the challenges of MSN, such as burst release and low circulation time, poor targetability and severe toxicity. To have a safe delivery system, the nanoparticle was then modified by RGD. In contrast to plain arsenic trioxide, the targeted liposomal encapsulated MSN (RGD-L-MSN) prolonged the AUC and half-life by 2.4 and 1.7 times. Additionally, in H22 tumor grafted mouse model, the nanocarrier amended the anti-cancer potential and targeting ability of the anti-cancer agent (Fei et al., 2017). Very recently, liposomes were prepared with the aim of encapsulating and improving the aqueous solubility of galbanic acid (GA), which is an effective anti-proliferative and anti-angiogenic compound. However, it fails to deliver significant results due to poor bioavailability. To further enhance the targeting effect, the nano-preparation was decorated with RGD peptide (Figure 6). The final formulation was successful at overcoming the solubility issues, due to entrapment of drug in the lipid bilayer. As compared to other preparations, the optimized liposome (size: 100 nm, zeta potential: -22.5, PDI: 0.16 with entrapment efficiency: 77%) showed that slow release is essential for the specific release of the tumor environment. The accumulation of targeted liposomal preparation was more than the non-targeted liposomes in HUVEC cells (colon cancer cells), which was due to tumor homing effect resulting from the interaction of RGD with integrin. The cell viability was less after treatment with RGD modified preparation compared to free galbanic acid. *In vivo* anti-tumor efficacy was also elevated after treatment with RGD-liposomes bearing galbanic acid (Nik et al., 2021). The outcome of the therapy proved to have strong anti-cancer effects.

## Conclusion and Challenges

Due to their versatile applicability, liposomes represent the most appealing delivery system. From enhancing the solubility of water insoluble drugs to their accumulation at tumor cells, liposomes have beneficial features. A simple modification of liposomes such as PEGylation or using pH or thermosensitive lipid accelerates their clinical demands and safety profile. RGD peptides demonstrate excellent ligand binding ability towards the integrin family and its modification with liposomes provides broad-spectrum therapy. Integrins act as cellular mechanotransducers, which are involved in various cancers such as breast, colon, lung, hepatic, and skin, etc.,

Activation of the integrin mediated bidirectional signaling feeds tumor growth and metastasis. Therefore, targeting integrin in cancer stroma is a crucial avenue for uncovering therapeutic options. In principle, liposomal therapy targets integrin via ligation with RGD peptide perpetuated high uptake, profound release, reduced cell viability, improved cytotoxicity, and elevated survival. Thus, inhibiting the activation of integrins at any stage of cancer could be beneficial for cancer patients. However, RGD engineered liposomes are still in the early stages of development for target mediated treatment. Several challenges such as non-specific binding with serum and recognition by the immune system disable their functions. To date, RGD modified liposomes have not received significant attention in clinical trials. The reason for this could be instability and low drug loading. Moreover, the high rate of cancer heterogeneity poses a major challenge in its utilization.

In general, *in vivo* cell specificity is somewhat different from *in vitro* cell targeting as an expression of integrin over endothelial cells. Further knowledge of their behavior in the cellular environment is critical and extensive molecular studies are required before jumping into therapy.
